# The *in vivo* and *in vitro* roles of *Trypanosoma cruzi* Rad51 in the repair of DNA double strand breaks and oxidative lesions

**DOI:** 10.1371/journal.pntd.0006875

**Published:** 2018-11-13

**Authors:** Danielle Gomes Passos Silva, Selma da Silva Santos, Sheila C. Nardelli, Isabela Cecília Mendes, Anna Cláudia Guimarães Freire, Bruno Marçal Repolês, Bruno Carvalho Resende, Héllida Marina Costa-Silva, Verônica Santana da Silva, Karla Andrade de Oliveira, Camila Franco Batista Oliveira, Liza Figueiredo Felicori Vilela, Ronaldo Alves Pinto Nagem, Glória Regina Franco, Andrea Mara Macedo, Sergio Danilo Junho Pena, Erich Birelli Tahara, Policarpo Ademar Sales Junior, Douglas Souza Moreira, Santuza Maria Ribeiro Teixeira, Richard McCulloch, Stela Virgilio, Luiz Ricardo Orsini Tosi, Sergio Schenkman, Luciana Oliveira Andrade, Silvane Maria Fonseca Murta, Carlos Renato Machado

**Affiliations:** 1 Departamento de Bioquímica e Imunologia, ICB, Universidade Federal de Minas Gerais, Belo Horizonte, MG, Brazil; 2 Centro de Pesquisas René Rachou/FIOCRUZ, Belo Horizonte, MG, Brazil; 3 Instituto Carlos Chagas/FIOCRUZ, Rua Professor Algacyr Munhoz Mader 3775, Curitiba, PR, Brazil; 4 Wellcome Trust Centre for Molecular Parasitology, Glasgow Biomedical Research Centre, University of Glasgow, Glasgow, Scotland, United Kingdom; 5 Department of Cell and Molecular Biology, Ribeirão Preto Medical School, University of São Paulo, Ribeirão Preto, São Paulo, Brazil; 6 Departamento de Microbiologia, Imunologia e Parasitologia, Universidade Federal de São Paulo, Rua Pedro de Toledo 669, São Paulo, São Paulo, Brazil; 7 Departamento de Morfologia, ICB, Universidade Federal de Minas Gerais, Caixa Postal 486, Belo Horizonte, MG, Brazil; Instituto de Investigaciones Biotecnológicas, ARGENTINA

## Abstract

In *Trypanosoma cruzi*, the etiologic agent of Chagas disease, Rad51 (TcRad51) is a central enzyme for homologous recombination. Here we describe the different roles of TcRad51 in DNA repair. Epimastigotes of *T*. *cruzi* overexpressing TcRAD51 presented abundant TcRad51-labeled foci before gamma irradiation treatment, and a faster growth recovery when compared to single-knockout epimastigotes for RAD51. Overexpression of RAD51 also promoted increased resistance against hydrogen peroxide treatment, while the single-knockout epimastigotes for RAD51 exhibited increased sensitivity to this oxidant agent, which indicates a role for this gene in the repair of DNA oxidative lesions. In contrast, TcRad51 was not involved in the repair of crosslink lesions promoted by UV light and cisplatin treatment. Also, RAD51 single-knockout epimastigotes showed a similar growth rate to that exhibited by wild-type ones after treatment with hydroxyurea, but an increased sensitivity to methyl methane sulfonate. Besides its role in epimastigotes, TcRad51 is also important during mammalian infection, as shown by increased detection of *T*. *cruzi* cells overexpressing RAD51, and decreased detection of single-knockout cells for RAD51, in both fibroblasts and macrophages infected with amastigotes. Besides that, RAD51-overexpressing parasites infecting mice also presented increased infectivity and higher resistance against benznidazole. We thus show that TcRad51 is involved in the repair of DNA double strands breaks and oxidative lesions in two different *T*. *cruzi* developmental stages, possibly playing an important role in the infectivity of this parasite.

## Author summary

*Trypanosoma cruzi* is the causative agent of Chagas disease, a tropical neglected illness that affects 6 million people worldwide, mostly in Latin America. Our research group focuses on the elucidation of DNA repair and metabolism of *T*. *cruzi*, and on the possible implications of those mechanisms in both parasite genetic diversity and Chagas disease development. In this work, we investigated the involvement of homologous recombination in the oxidation-induced damage response, and in DNA repair, in *T*. *cruzi* cells after gamma radiation exposure. We also examined whether TcRad51 –a key protein for homologous recombination–could be an important factor for *T*. *cruzi*’s infectivity. We generated genetically-modified *T*. *cruzi* cells for RAD51 and studied the *in vitro* and *in vivo* infectivity of these mutant cells. Our results indicate that TcRad51 is a key protein involved in the repair of oxidative and DNA double-strand breaks lesions in two crucial developmental forms of *T*. *cruzi*.

## Introduction

Homologous recombination (HR) is a crucial, error-free pathway required to repair a diverse array of potentially lethal lesions, which include interstrand crosslinks, ssDNA gaps left behind by the replication fork, and DNA double-strand breaks (DSBs)–generated either as a result of replication fork collapse, or from processing of spontaneous damage or from exposure to DNA-damaging agents such as gamma radiation. Among these lesions, DSBs present the highest cytotoxicity, since a single unrepaired DSB can lead to aneuploidy, genetic aberrations, and cell death [1,2].

Rad51 is a key component of the HR machinery. This protein binds exposed 3’ single strand DNA ends at a DSB, and catalyzes their invasion into intact DNA duplex, generating homologous pairing and promoting recombination [3]. Studies in yeast, mammalian cells, and other organisms revealed a role for Rad51 in the repair of DSBs generated upon treatment with ionizing radiation and other genotoxic agents [4–7]. The process of HR has been described in trypanosomatids, a group of protozoa involved in several human and animal parasitic diseases like Chagas disease, sleeping sickness, and leishmaniasis. African trypanosomes use DNA recombination to evade the mammalian immune system through antigenic variation; in fact, mutation of Rad51 from *Trypanosoma brucei* impairs HR, reduces the frequency of antigenic variation, and increases the parasitic sensitivity to DSBs-inducing agents [6]. In most eukaryotes, the major alternative repair mechanism of DSBs is the non-homologous end-joining (NHEJ) pathway. Genome sequences of *T*. *brucei*, *Trypanosoma cruzi* and *Leishmania major* revealed that trypanosomatids appear to lack some of the core proteins for NHEJ [8]. Experiments in *T*. *brucei* designed to detect NHEJ supported its absence and revealed an end-joining process mediated by sequence microhomology, suggesting that HR plays the predominant role in the repair of DSBs [9,10]. Glover and colleagues (2008) reinforced this idea when demonstrated that the repair of a single DSB in the genome of *T*. *brucei* is preferentially performed by HR through foci formation promoted by Rad51 [11], and cell cycle arrest. Rad51 from *L*. *major* also seems to present a role in DSBs repair since treatment with phleomycin causes an elevation in the transcription of this gene [12]. Interestingly, little work has been done to study HR in *T*. *cruzi*, the causative agent of Chagas disease, a debilitating illness that affects 10 million people worldwide, mostly in Latin America. Alarmingly, 25 million people are currently exposed to the risk of this disease [13].

*T*. *cruzi* is a parasite that has two hosts during its life cycle: one invertebrate, which is an insect of the *Reduviidae* family, and the other vertebrate, which comprises several mammalian species, including humans. Having such a complex life cycle, *T*. *cruzi* has to present adaptability to very distinct environments. During its insect phase, this parasite grows in the intestine lumen, where it is exposed to several environmental changes such as temperature, osmolarity, pH, nutrients and oxidative stress, both from hemoglobin degradation and nitrogen intermediary production from the insect immune system [14–16]. Infection of mammalian cells begins with the recruitment and fusion of lysosomes, organelles that present a highly acidic, oxidative environment. Thereafter, a parasitophorous vacuole is formed in the host cell from which the parasite is able to escape towards the cytoplasm. Then, *T*. *cruzi* cells divide as amastigotes, which are a target of reactive oxygen species produced by the host cell and its respiratory chain [17,18].

Our first step to better understand the molecular mechanisms of HR in *T*. *cruzi* was to characterize TcRad51, whose mRNA is shown to be transcribed in all three life cycle forms of *T*. *cruzi* [7]. Remarkably, when we examined the resistance of *T*. *cruzi* epimastigotes against gamma irradiation (1–5 kGys), we found that this genotoxic treatment resulted in cellular growth arrest, and also in DNA fragmentation, resulting in shattered chromosomes. Shattered chromosomes were repaired in 48 h, and epimastigotes overcame the growth arrest a few days later. A direct role for TcRad51 in DSBs repair was suggested by the induction of TcRad51 gene expression after the exposure of epimastigotes to gamma rays. Indeed, overexpression of TcRad51 in *T*. *cruzi* resulted in a faster kinetics of chromosome repair, with intact chromosomes becoming visible within 24 h, leading to a faster cell growth recovery [7]. RAD51-overexpressing cells (TcRAD51^ox^) also presented higher resistance against zeocin, another DSBs-inducing agent. To further understand the role of TcRad51 in the repair of DSBs in *T*. *cruzi*, we generated single TcRAD51 allele knockout cells (TcRAD51^+/-^) through targeted gene deletion of TcRAD51. We compared the TcRAD51^+/-^ knockout cells to both TcRAD51^ox^ and wild-type (WT) cells after exposing them to different genotoxic agents: gamma radiation, UV light, cisplatin, hydroxyurea (HU) and MMS. Since these agents cause distinct lesions, our findings show that the levels of TcRad51 dictate the sensitivity of parasites to DSBs and oxidative agents, but not to cross-linking agents. Additionally, we showed that TcRad51 probably acts as a contributing factor for infectivity in *T*. *cruzi*, since TcRAD51^ox^ cells present an increased number of parasites in fibroblasts and macrophage cells when compared to WT parasites. TcRAD51^ox^ cells also presented an increased parasitemia and greater resistance against benznidazole in animal models.

## Materials and methods

### Ethics statement

All mouse experiments were performed in compliance with the NIH Guide for the Care and Use of Laboratory Animals and were approved by the Institutional Animal Care and Use Committee at the Centro de Pesquisas René Rachou/FIOCRUZ (CEUA-LW-54-12, Centro de Pesquisas René Rachou/FIOCRUZ).

### Cellular growth

Epimastigotes from *T*. *cruzi* clone CL Brener were cultivated at 28°C in liver infusion tryptose (LIT) medium (pH 7.3) supplemented with 10% fetal calf serum (FCS), streptomycin sulfate (0.2 mg.mL^-1^) and penicillin (200 units.mL^-1^). WT cells, as well as TcRAD51^ox^ cells, were characterized by Regis-da-Silva *et al*. (2006) [7].

### Plasmid construction and transfection

Vectors used to delete TcRAD51 were generated as follows: primers were designed to amplify DNA fragments of 461 and 541 bp of length from the 5’UTR and 3’UTR of TcRAD51, respectively. These two resultant DNA fragments were designed to flank the hygromycin phosphotransferase gene to obtain, through cloning using a pGEM-T vector, the deletion cassette harboring ApaI and SacI sites. Resultant constructs were released by ApaI and SacI digestion and then transfected into electroporated *T*. *cruzi* epimastigotes according to a previously described protocol [19]. Transfected parasites were selected after 4 weeks of culture in the presence of hygromycin B (200 μg.mL^-1^). Isolated hygromycin B-resistant clones were obtained as previously described in [20].

### Real-time PCR

Genomic DNA from WT and modified *T*. *cruzi* strains were extracted using the PureLink Genomic DNA Mini Kit (ThermoFisher Scientific), using the indicated protocol from the manufacturer. DNA integrity was verified by running the samples onto an electrophoresis gel, and 5 ng of total DNA were used for real-time PCR. The number of TcRAD51 copies in each strain was determined by using genomic DNA as a template for reactions containing 5 μL SYBR Green PCR Master Mix (Applied Biosystems) with 0.25 M of each primer (TcFwRAD51: GTGCCCTCGTGGTAAACC and TcRevRAD51: GCGGATGAACCCATT). Reactions were performed using Applied Biosystems 7900HT Fast Real-Time PCR System. Reaction conditions are already described in a previous study [7].

### Southern blotting analysis

Genomic DNA (5 μg) from *Trypanosoma cruzi* WT and RAD51+/- cells were treated with *Nde*I and *Bgl*I restriction endonucleases at 37°C for 24 h and digestion products were resolved by gel electrophoresis (0.7% agarose; 1x TAE; at 40V; for 16 h). DNA was transferred to Hybond-N+ membranes (GE Life Sciences) and probed with PCR-amplified 1062 bp hygromycin (HYG) and ~550 bp 3’-RAD51 (RAD) fragments from *T*. *cruzi*. Hybridization was carried out at 60°C using Amersham^TM^ AlkPhos Direct Labelling and Detection System with Amersham^TM^ CDP-Star^TM^ Detection Reagent (GE Life Sciences).

### Anti-TcRad51 antiserum and Western blotting

Recombinant TcRad51, purified as a C-terminally His-tagged variant in *Escherichia coli*, was used to immunize mice in order to obtain anti-TcRad51 antiserum. C57Bl/6 mice were immunized with 10 μg of TcRad51-His protein in aluminum hydroxide adjuvant by subcutaneous injection. Four subsequent boosts (15, 30, 45 and 60 d after the first immunization) were performed. Mouse serum was collected 10 d after the last immunization, and non-immune serum was obtained from the same mouse before first immunization.

For Western blot assays, exponentially grown *T*. *cruzi* epimastigotes were used to prepare protein extracts. Cells were washed and resuspended in SDS gel-loading buffer [100 mM Tris-HCl (pH 6.8), 200 mM dithiothreitol, 4% SDS, 0.2% bromophenol blue, 20% glycerol] at a final concentration of 2.10^5^ cells, and boiled for 10 min, generating the total extract. Proteins were separated on a 10% SDS polyacrylamide gel, blotted (2 h, 200 mA) onto nitrocellulose membranes, and incubated with primary antibody [1:2,000 dilution for anti-TcRad51; 1:3000 for rabbit anti-γH2A, produced and kindly provided by Dr. Richard McCulloch’s lab [21]; or 1:12,000 dilution for anti-alpha-tubulin monoclonal (Abcam)], and then with secondary antibody conjugated with peroxidase [anti-mouse or rabbit IgG (GE), 1:10,000 dilution]. Antibody binding was visualized through the use of ECL-Plus Western Blot Detection System (GE). Images were recorded using STORM Phosphoimager (GE).

### TcRad51 immunolocalization

TcRad51 immunolocalization experiments were performed by attaching 2.10^5^ cells prewashed with PBS onto glass slides, and fixation was carried out with 4% *p*-formaldehyde in PBS for 20 min. Slides were then washed three times with PBS and treated with 0.1% Triton X-100 in PBS for 5 min, before being incubated for 30 min with 1% bovine serum albumin diluted in PBS. Next, slides were incubated overnight with polyclonal anti-TcRad51 antiserum diluted 1:2,000 in PBS containing 1% bovine serum albumin and, after that, were washed three times. Primary antibodies were detected with anti-mouse secondary antiserum conjugated with Alexa Fluor 555 (anti-mouse IgG–Invitrogen) diluted 1:5,000 in the presence of 0.01 mM 4',6-diamidino-2-phenylindole (DAPI). Vectashield (Vector Laboratories) was used for mounting, and slides were visualized using a 100X oil immersion objective (1.3 numerical aperture) from a Nikon E600 microscope coupled to a Nikon DXM1200F camera. Images were processed for color using Adobe Photoshop software (Adobe Systems Incorporated). At least 200 cells were analyzed per coverslip.

Fluorescence quantification was performed using ImageJ software (http://rsb.info.nih.gov/ij/). For each time point after gamma radiation, approximately thirty parasites were analyzed. Nuclei were individually delimited, and the mean labeling intensity under sub-saturating conditions was determined. Student’s *t*-test and one-way analysis of variance (ANOVA) followed by a Bonferroni post-test were performed using GraphPad Prism software (version 3.0).

### Genotoxic treatment

WT epimastigotes were compared to TcRAD51^ox^ cells and to TcRAD51^+/-^ parasites by plating 1x10^7^ cells.mL^-1^ in the presence or absence of genotoxic agents. For hydrogen peroxide (H_2_O_2_) treatment, parasite cultures were treated with 0, 150, 200 or 250 μM H_2_O_2_. UVC irradiation (254 nm) was performed with a germicidal lamp at a fluence rate of 0, 5,000, 10,000 or 15,000 J.m^-2^. For cisplatin treatment, cells were incubated with 0, 25, 50 or 75 μM cisplatin. In all aforementioned conditions, surviving cells were counted 2 d after treatment. For HU treatment, parasites were incubated with 0, 10, 20 or 30 mM HU for 24 h in LIT medium. For MMS treatment, parasites were incubated with 1.5 mM MMS for 60 min in PBS buffer. For HU and MMS treatments, drugs were removed by centrifugation, and cells were incubated in fresh LIT medium. The number of parasites was determined every 24 h, for 5 d. For gamma irradiation treatment, cell cultures were exposed to a dose of 1578 Gy.h^-1^ for 20 min using a cobalt (^60^C) irradiator (Gamma Radiation Laboratory–CDTN, UFMG). The number of cells was determined for a period of 16 d. Erythrosine vital stain assay using a cytometry chamber was the method used to differentiate living and dead cells. Experiments were performed in triplicate.

### Pulsed-field gel electrophoresis

Chromosomes from different *T*. *cruzi* samples (CL Brener WT, TcRAD51^ox,^ and TcRAD51^+/-^) were treated with gamma irradiation (500 Gy) as described above and incubated in LIT medium for 0, 24, 48, 72 or 96 h. After, agarose blocks containing intact *T*. *cruzi* chromosomes were prepared and separated by pulsed-field gel electrophoresis (PFGE) using the Gene Navigator TM system (GE Life Sciences, Little Chalfont, UK) as previously described [22]. Optimized separations of chromosomes were obtained using pulsed-field gel electrophoresis intervals of 90 s for 30 h, 200 s for 30 h, 400 s for 30 h and 600 s for 18 h at 90V and 9°C. Following PFGE, gels were stained with ethidium bromide (0.5 μg.mL^-1^).

### *In vitro* cell infection experiments

All *in vitro* cell infection experiments were performed using a mouse fibroblast cell lineage (WTCl3) derived from mouse embryonic fibroblasts [23]. Prior to infection, fibroblasts were plated at the concentration of 2.5.10^4^ cells/mL in medium containing 10% FBS in 24-well tissue culture plates containing 12 mm round coverslips, and grown for 24 h, at 37°C, in a humidified atmosphere containing 5% CO_2_. Infection of fibroblasts with purified tissue culture trypomastigotes (TCTs) was performed for 30 min at 37°C at a multiplicity of infection (MOI) of 50. Immediately after infection, fibroblasts were washed four times with PBS in order to remove extracellular parasites, and re-incubated with fresh medium for 30 min (time point = 0 h), 24, 48, 72 and 96 h, before fixation with 4% (wt/vol) paraformaldehyde/PBS overnight, at 4°C.

After fixation, coverslips with attached fibroblasts were washed three times in PBS, incubated for 20 min with PBS containing 2% BSA, and processed for an inside/outside immunofluorescence invasion assay as described previously [24]. Next, the DNA of host cells and parasites was stained for 1 min with DAPI (Sigma), mounted, and examined on a Zeiss Axioplan microscope (Carl Zeiss AG). Approximately 200 to 300 fibroblasts were counted, and 15 to 20 fields were evaluated per coverslip.

### Mice infection and parasitemia curves

Blood of mice infected with *T*. *cruzi* was collected from orbital venous sinus (0.5–0.6 mL), diluted in 3.8% sodium citrate, and inoculated intraperitoneally in non-infected mice. Groups of 19 to 22 male Swiss albino mice of 18–20 g were inoculated with 5,000 bloodstream forms of each *T*. *cruzi* sample. Parasitemia was followed from the 5^th^ to the 22^nd^ day of infection by collecting fresh blood from the tail of mice, and estimating the number of parasites as described by Brener (1962) [25]. Parasitemia curves and the mortality rate of mice infected with different *T*. *cruzi* samples were evaluated.

### Analysis of susceptibility of long-term benznidazole-treated mice

Groups of 12 or 14 male Swiss mice ranging from 18 to 20 g were intraperitoneally inoculated with 5,000 *T*. *cruzi* blood forms. Five days after inoculation, every animal was examined in order to ensure that the infection was properly established. Then, *T*. *cruzi*-infected mice were submitted to a long-term treatment with oral doses of benznidazole (100 mg/kg of body mass) for 20 consecutive days. Hemoculture was used as the criterion of cure for benznidazole-treated mice. Thirty days after the end of long-term benznidazole treatment, mice were the subject of orbital venous sinus bleeding, and 0.6 mL of blood was collected and divided into 2 tubes containing 5 mL of LIT medium. Tubes were incubated at 28°C for 30 to 60 days and examined microscopically in order to verify the possible presence of parasites. If both hemoculture tubes were negative for *T*. *cruzi*, the animal was considered cured.

### Immunofluorescence of parasite infection

After fixation, coverslips with attached cells were washed three times in PBS, incubated for 20 min with PBS containing 2% bovine serum albumin (PBS/BSA), and processed for an inside/outside immunofluorescence invasion assay as described previously [24]. Briefly, to distinguish extracellular parasites from intracellular ones, cells were incubated for 50 min in the presence of a polyclonal anti-TcRad51 (produced in rabbit) solubilized in PBS/BSA (1:500 dilution), followed by a 40-min incubation with Alexa-Fluor 546 goat anti-rabbit IgG (Invitrogen) diluted 1:250 in PBS/BSA. For vacuole escape kinetics experiments, after extracellular-parasite staining, cells were permeabilized with PBS/BSA containing 0.5% saponin (PBS/BSA/saponin) for 20 min. Parasites associated with parasitophorous vacuole were labeled using a 50 min-incubation with rat anti-LAMP1 antibody diluted 1:50 in PBS/BSA/saponin, followed by 40 min-incubation with a 1:250 dilution of Alexa-Fluor 488 goat anti-rat IgG (Invitrogen) in PBS/BSA/saponin. DNA from cells and parasites was stained with DAPI diluted 1:1,000. Slides were mounted and then examined on a Zeiss Axioplan-2 microscope. Experiments were performed in biological triplicates, and at least 200 cells were analyzed per coverslip.

### Macrophage infection experiments

For experimental infection of THP-1 macrophages, cells were diluted to a density of 5.10^5^ cells.mL^-1^ in RPMI medium supplemented with 10% FBS. Immediately after the addition of propidium monoazide (PMA) at a concentration of 50 mg.mL^-1^, macrophages were seeded onto 24-well plates (containing round glass coverslips of 13 mm diameter), and incubated at 37°C in an atmosphere with 5% CO_2_ for 72 h to allow differentiation. Tissue-culture trypomastigotes were then purified, counted and diluted in RPMI medium, and infection was performed for 2 h at an MOI of 5. Immediately after macrophage infection, cells were washed four times with PBS to remove extracellular parasites, and then either fixed or re-incubated with medium for 48 and 72 h before fixation with methanol. Coverslips with attached macrophages were stained with Giemsa, and a minimum of 300 macrophages per coverslip were analyzed. Results were expressed as an infection index (% infected macrophages vs. number of amastigotes per the total number of macrophage). Experiments were performed in triplicate.

### Statistical analysis

Statistical analyses were performed using the GraphPad Prism 5.0 (GraphPad Software Inc., CA, USA). Data are presented as mean±standard deviation (SD). The level of significance was set to *p* < 0.05. All experiments were performed at least three times.

## Results

### Generation of TcRad51 single knockout cells

**Fig 1:**
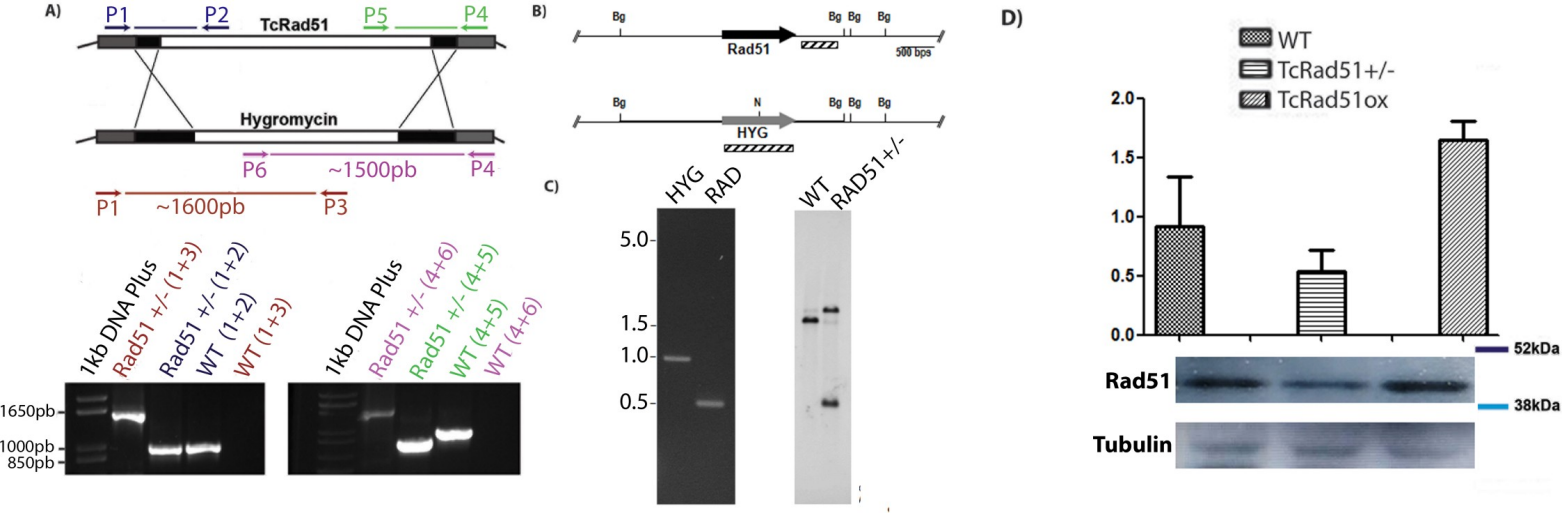
Generation of TcRAD51^-/+^ knockouts mutants and TcRad51 protein levels. (A) Ethidium bromide stained gel showing PCR products generated with primers represented indicated in the schematic representation. PCR was performed using genomic DNA of WT and TcRAD51^+/-^ epimastigotes. Upper panel: schematic representation of RAD51 and hygromycin allele detection. Note that the amplicon is only synthesized if the pair of primers simultaneously hybridizes inside and outside the deletion cassette. The figure is not on scale. (B) Schematic representation of the Southern blot analysis showing BglI sites and the probes used (C) Left panel: Purified HYGB and RAD51 fragments generated by PCR-amplification. Right panel: Southern blot analysis of BglI- and BglI-digested genomic DNA from WT and TcRAD51^+/-^ cells and probed with HYGB and RAD51 fragments. Ethidium bromide-stained agarose gel showing the digestion products that were further analyzed by Southern blotting. (D) Detection of TcRad51 levels in epimastigotes protein extracts from WT, TcRAD51^ox,^ and TcRAD51^-/+^ cells. Cellular lysates were separated by SDS-PAGE, and proteins were detected by Western blot with anti-TcRad51 (1:2,000) antiserum and peroxidase-conjugated anti-IgG secondary (1:10,000 or 1:12,000). A control showing tubulin levels was performed using mouse anti-tubulin (1:12,000) antiserum.

**Fig 2:**
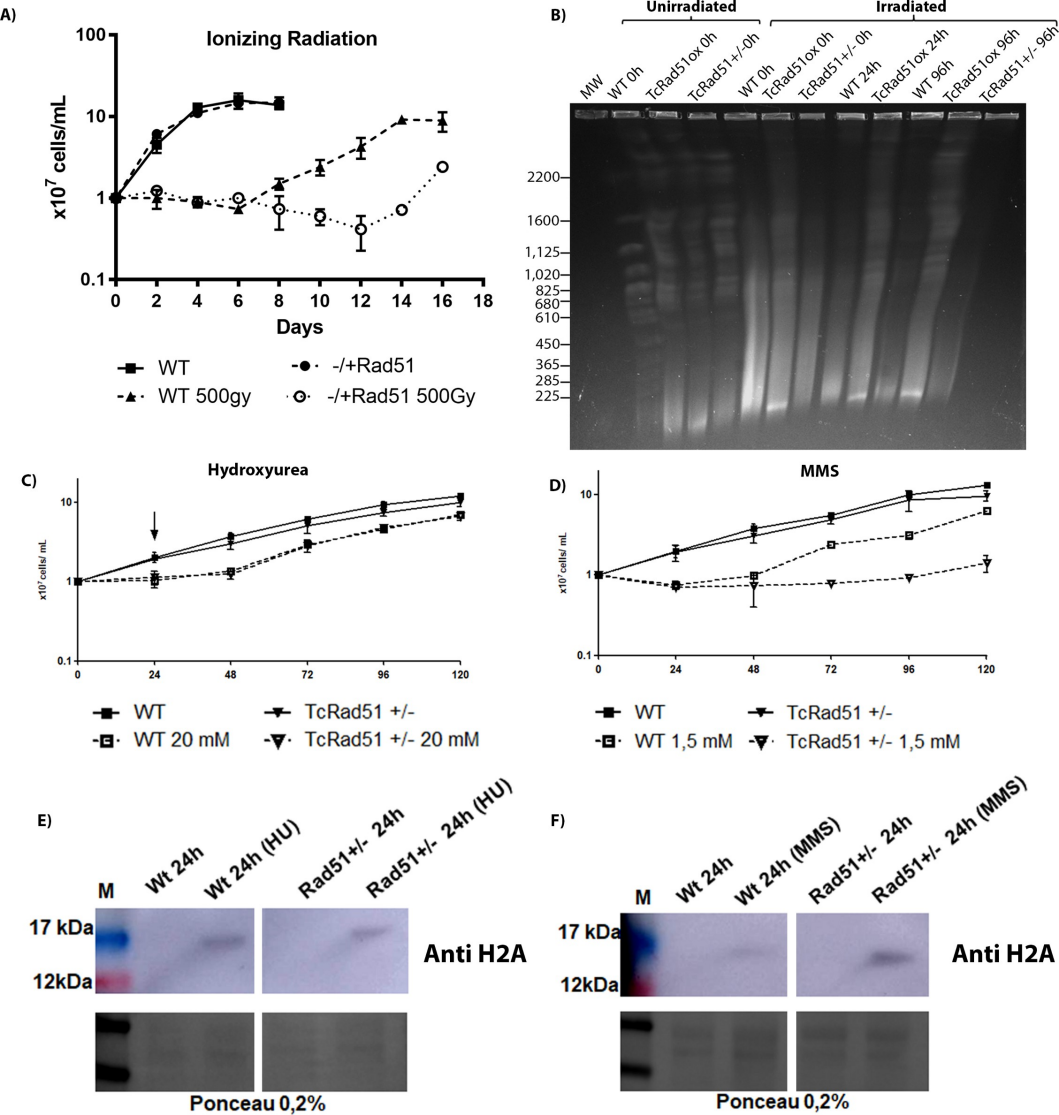
Growth of *T*. *cruzi* following DNA damage treatment. A) The sensitivity of WT and TcRAD51^-/+^ cells to gamma radiation was determined. Parasites were exposed to 0 Gy or 500 Gy of gamma irradiation, the number of parasites was determined every two days for 28 d. Values represent the mean of triplicates and error bars indicate standard deviations. B) Chromosomal profile from different *T*. *cruzi* (WT, TcRAD51^ox,^ and TcRAD51^+/-^) samples after treatment with gamma radiation (500 Gy). Chromosomal bands from different *T*. *cruzi* samples were separated by PFGE and stained using ethidium bromide. Whole chromosomes from *Saccharomyces cerevisiae* were used as molecular weight markers. (C) *T*. *cruzi* growth curve after treatment with 20 mM HU. Arrow indicates the point when the drug was removed. (D) *T*. *cruzi* growth curve after treatment with 1.5 mM MMS. The number of cells was determined every 24 h by vital staining. Curves shown are an example of three independent experiments that were performed in triplicate. Error bars represent standard deviations. (E) Detection of H2A levels in epimastigotes treated with 20 mM HU. Cell lysates were separated by SDS-PAGE, and proteins were detected by Western blot using anti-H2A (1:3,000) antiserum and peroxidase-conjugated anti-IgG secondary (1:10,000 or 1:12,000). Loading control is depicted in the bottom panel (Ponceau 0.2%). (F) Detection of H2A levels in epimastigotes treated with 1.5 mM MMS. Cell lysates were separated by SDS-PAGE, and proteins were detected by Western blot with anti-H2A (1:3,000) antiserum and peroxidase-conjugated anti-IgG secondary (1:10,000 or 1:12,000). Loading control is depicted in the bottom panel (Ponceau 0.2%).

In order to evaluate the roles of TcRad51, we disrupted one allele of the TcRAD51 gene (Gene ID: 3546453 and 3537322) in the *T*. *cruzi* genome. As described in *Experimental procedures*, a TcRAD51 deletion cassette was obtained and used to transfect epimastigote cells. *T*. *cruzi* hygromycin B-resistant clones were then recovered (sequence analysis is shown in S1 Fig). PCR analysis showed that one allele of TcRAD51 was disrupted in the selected clones (Fig 1A). The single allele deletion confirmation was conducted by real-time PCR analysis with genomic DNA of WT and TcRAD51^+/-^ cells, allowing the verification that the CT value for TcRAD51 amplification was 20.89 in TcRAD51^+/-^, while 18.49 for WT cells–TcATR was used as control, and its CT value was 19.89 in TcRAD51^+/-^, and 20.01 in WT cells. The Southern blot analysis using a probe which hybridizes with hygromycin B resistance gene (Fig 2B, lower panel) showed two bands of increased intensity (~1300 and ~2200 bp) when DNA from TcRAD51+/- was assayed (Fig 1C, Probe HYG), resulted from the digestion of the hygromycin B-mutated allele with both BglII and NdeI–in fact, the hygromycin B resistance gene displays a NdeI restriction site (Fig 1B). The faint band observed around 3500 bp in the TcRAD51+/- lane is probably due to incomplete digestion of genomic DNA. As expected, WT cells do not harbor the hygromycin B resistance gene and thus not produce any signal. Besides that, when a probe which hybridizes with TcRAD51 was used (Fig 1B, upper panel), the same band of ~1300 was observed when DNA from TcRAD51+/- was assayed, indicating the presence of a DNA fragment double-digested with BglII and NdeI, leading to the conclusion that the hygromycin B resistance gene was successfully inserted into the TcRAD51 locus. Also, in line with predictions, a larger fragment produced from the digestion with BglII was produced in both WT and TcRAD51^+/-^ cells, indicating the existence of a WT locus of TcRAD51. Also, interestingly, the two larger bands (~3400 and 3600 bp) produced by BglII digestion when using the TcRAD51-hybridizing probe in WT cells did not display the same signal intensity which was expected (Fig 1C, Probe RAD)–the genome sequence of *T*. *cruzi* in TriTrypDB indicates the presence of two alleles of TcRAD51 that differs by a DNA insertion in a non-transcribed region (S1 Fig). Since the ~3400 bp fragment is the one most present in the WT cells, we infer that the majority of the WT CL Brener population used for the experiments is homozygous for the non-Esmeraldo TcRAD51 allele. Also, the Southern blot results (Fig 1C), together with the genomic DNA sequencing (S1 Fig), indicates that the hygromycin B resistance gene was inserted into the TcRAD51 locus of a *T*. *cruzi* population that is homozygous for the Esmeraldo TcRAD51 allele. We speculate that, after the selection pressure, a specific subpopulation of CL Brener, homozigous for the TcRAD51 alelle, was transformed with the DNA cassete and, therefore, generated the pattern observed on Fig 1.

### Levels of TcRad51 affect *T*. *cruzi* growth and DNA repair after gamma irradiation

In a previous report, we showed that overexpression of TcRAD51 leads to an increased efficiency of DNA repair of shattered chromosomes caused by gamma irradiation [7]. To further examine this observation, we asked if decreasing the levels of TcRad51 would promote an effect on the recombination levels in *T*. *cruzi*. We compared TcRAD51^+/-^ and WT cells after parasite exposure to 500 Gy of gamma radiation (Fig 2). Despite presenting similar growth rates in the absence of induced DNA damage, cells presented a distinct pattern of growth after exposure to gamma radiation. Immediately after irradiation, both WT and TcRAD51^-/+^ cells ceased dividing, although the number of cells did not decrease significantly. In WT, cells growth arrest persisted for 6 d, by the end of which cellular division resumed. In contrast, TcRAD51^+/-^ cells presented a considerable delay in growth recovery, with an increase in cell concentration only observed around 12 d after gamma irradiation. Indeed, the number of cells appeared to decrease to some extent until day 12 after induced DNA damage. A PFGE was performed, and chromosomes from WT, TcRAD51^ox,^ and TcRAD51^+/-^, after treatment with gamma radiation (500 Gy), were clearly separated and ranged in size from 225 to 2,200 Kb (Fig 2B). Chromosomal profile differed among *T*. *cruzi* samples analyzed after gamma irradiation. Interestingly, TcRAD51^ox^ parasites rapidly repair their chromosomes, presenting visible intact DNA content 24 h after exposure to gamma radiation; in contrast, TcRAD51^+/-^ parasites do not repair their chromosomes even 96 h after being exposed to gamma radiation. WT parasites repair their chromosomes since chromosome bands are observed after 96 h of gamma irradiation. These results confirm that the overexpression of TcRAD51 increases the resistance against gamma irradiation and that one TcRAD51 allele is sufficient to increase sensitivity to this genotoxic agent. Taken together, these results indicate a major role of TcRad51 in the repair of DSBs in *T*. *cruzi* DNA.

To investigate the role of TcRad51 in the repair of HU-induced replicative stress in WT cells and TcRAD51^+/-^ mutants, *T*. *cruzi* cells were treated with 20mM of this agent for 24 h (Fig 2C). No difference was observed when we used other doses of HU (S2 Fig). Both cell types exhibited the same growth profile, which suggests that TcRad51 is not important for the repair of this kind of DNA damage. Methylating agents, like MMS, produce several types of DNA adducts, which induce replication-dependent DSBs [26]. To study the role of TcRad51 in the repair of MMS-induced DNA damage, WT and TcRAD51^+/-^ cells were treated with 1.5 mM MMS for 60 min. TcRAD51^+/-^ cells showed a delayed recovery after the treatment in relation to WT cells (Fig 2D). In order to verify if the generation of DSB’s resulted from the exposure to MMS and HU, a Western blot was performed with the same amount of drugs used in the curves: both MMS and HU treatments were able to increase H2A, a marker for double-strand breaks, in WT cells and TcRAD51^+/-^ mutants (Fig 2E and 2F). Interestingly, the MMS treatment led to an increased amount of H2A in TcRAD51^+/-^ cells when compared to the WT strain; this result suggests that TcRad51 is necessary to cope with replicative stress generated by MMS. Since the level of H2A does not differ between WT cells and TcRAD51^+/-^ mutants after HU treatment, we suggest that TcRad51 is not involved in the repair of lesions promoted by HU.

### Kinetics of TcRad51 immunolocalization after exposure to gamma radiation

**Fig 3:**
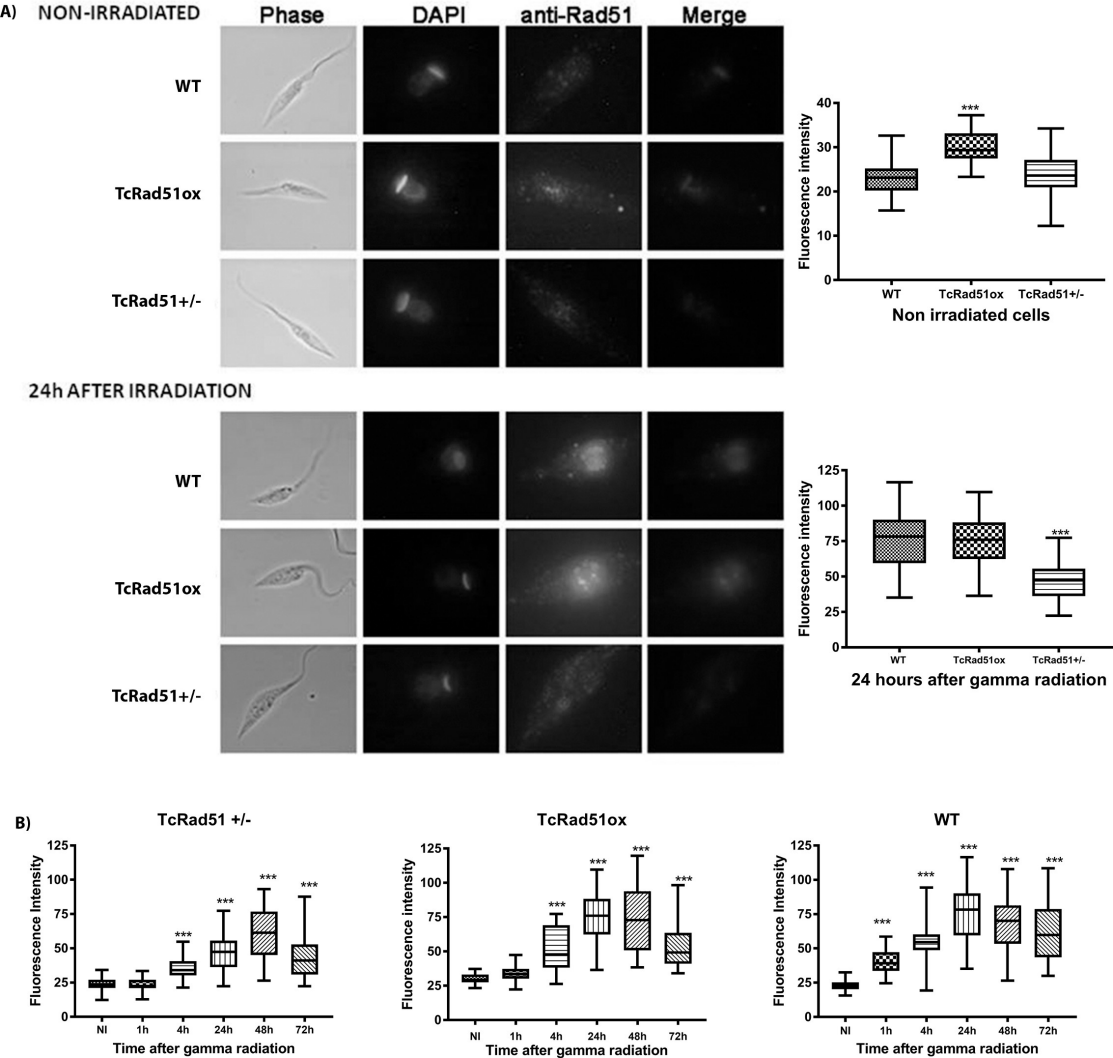
TcRad51 immunolocalization in *T*. *cruzi*. A) WT, TcRAD51^+/-^ and TcRAD51^ox^ cells are shown before irradiation (non-irradiated) and 24 h after exposure to 500 Gy of gamma irradiation. DNA is shown stained with DAPI and TcRad51 was detected using anti-TcRad51 antibody raised in mouse (diluted 1:2,000) and visualized with Alexa 555 conjugated goat-derived anti-mouse IgG secondary (diluted 1:5,000); black bar: 5 μm. Cells are also shown in phase contrast but at a lower magnification (white bar: 2 μm). A graphical representation of fluorescence intensities is shown alongside the images. Vertical bars indicate standard deviation, asterisks represent statistically significant differences (***p < 0.001, Student's *t*-test, Mann-Whitney post-test) in fluorescence between each time point comparing irradiated to non-irradiated parasites. At least 200 cells were analyzed per coverslip. B) Box-plot of fluorescence intensity of TcRad51 in the nucleus of WT, TcRAD51^-/+,^ and TcRAD51^ox^ cells are shown before irradiation (non-irradiated) and at different times (1, 4, 24, 48 and 72 h) after 500 Gy of gamma irradiation exposure. Two hundred cells were analyzed per coverslip. Asterisks represent statistically significant differences (***p < 0.001, *t-student* test with Mann-Whitney post-test) in fluorescence between each time point comparing irradiated to non-irradiated parasites.

To investigate the kinetics of TcRad51 function in the repair of DSBs in *T*. *cruzi*, we performed the immunolocalization of TcRad51. WT, TcRAD51^+/-^ and TcRAD51^ox^ cells were analyzed at different times (0, 1, 4, 24, 48 and 72 h) after exposure to 500 Gy of gamma radiation. Fig 3A shows a low detectable TcRad51 signal prior to irradiation in WT and TcRAD51^+/-^ cells compared to visible foci seen in TcRAD51^ox^ parasites. Following the damage, a progressive accumulation of TcRad51 was observed in the nucleus, with discreet foci formation after 24 h of damage, as previously shown for *T*. *brucei* after phleomycin treatment [27]. A less intense staining accumulation throughout the nucleus was verified for TcRAD51^+/-^ cells in comparison to the other cell lines. TcRAD51^ox^ cells presented higher fluorescence intensity before radiation compared to WT cells, though maximal signal levels were similar for both of them 24 h after irradiation (Fig 3A, lower panel). We were able to detect TcRad51 in the nuclei of parasites at all time points analyzed (Fig 3B). TcRad51 fluorescence signal reached the highest level after 24 h and began to decrease thereafter in WT and TcRAD51^ox^ cells (Fig 3B middle and right panels). S3 Fig shows fluorescence images representing nuclei localization over time in all cells after gamma irradiation. In contrast, TcRAD51^+/-^ cells presented a delay in TcRad51 accumulation in the nucleus after radiation, with maximum signal only apparent after 48 h (Fig 3B); in addition, in these parasites, the levels of fluorescence were not comparable to those observed in WT cells. Taken together, these data suggest that decreasing or increasing the levels of TcRad51 result in changes in the kinetics of localization of this protein in the nucleus after radiation damage.

### TcRad51 does not mediate resistance against DNA cross-linking agents

**Fig 4:**
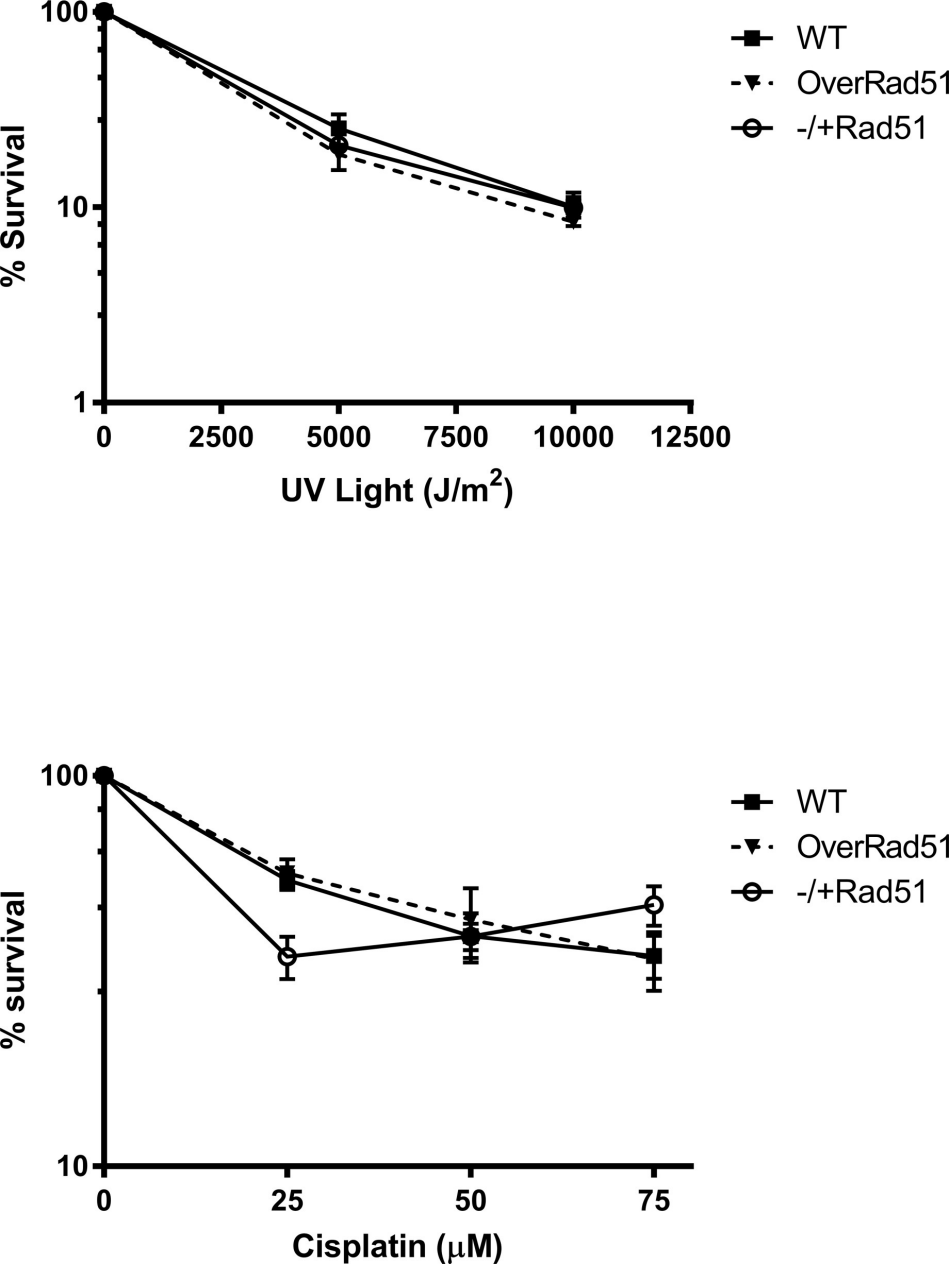
*T*. *cruzi* response to treatment with DNA cross-linking agents. Sensitivity of WT, TcRAD51^ox^ and TcRAD51^-/+^ cells to: (A) 0, 500 J/m^2^ or 1.000 J/m^2^ of UV-light, or (B) 25 μM, 50 μM or 75 μM cisplatin. Parasites were counted 48 h after treatment. Numbers are represented as a percentage of untreated cells. Values represent the mean of triplicates. Error bars indicate standard deviations.

Besides its role in the repair of DSBs, Rad51 –through homologous recombination–allows the restart of blocked replication forks [28]. In order to complete replication, cells must overcome replication fork barriers, such as secondary structures on DNA, or cross-links caused by treatment with genotoxic agents such as cisplatin and UV light. To verify whether TcRad51 plays a role in these processes, WT, TcRAD51^ox^ and TcRAD51^+/-^ cells were exposed to increasing doses of cisplatin or UV light (Fig 4A e 4B). All cells presented similar survival curves after both treatments. These results demonstrate that TcRad51 does not play a pivotal role in the resistance against agents involved in the generation of intra- or inter-strand DNA cross-link damage in *T*. *cruzi*.

### TcRad51 is involved in the repair of oxidative lesions

**Fig 5:**
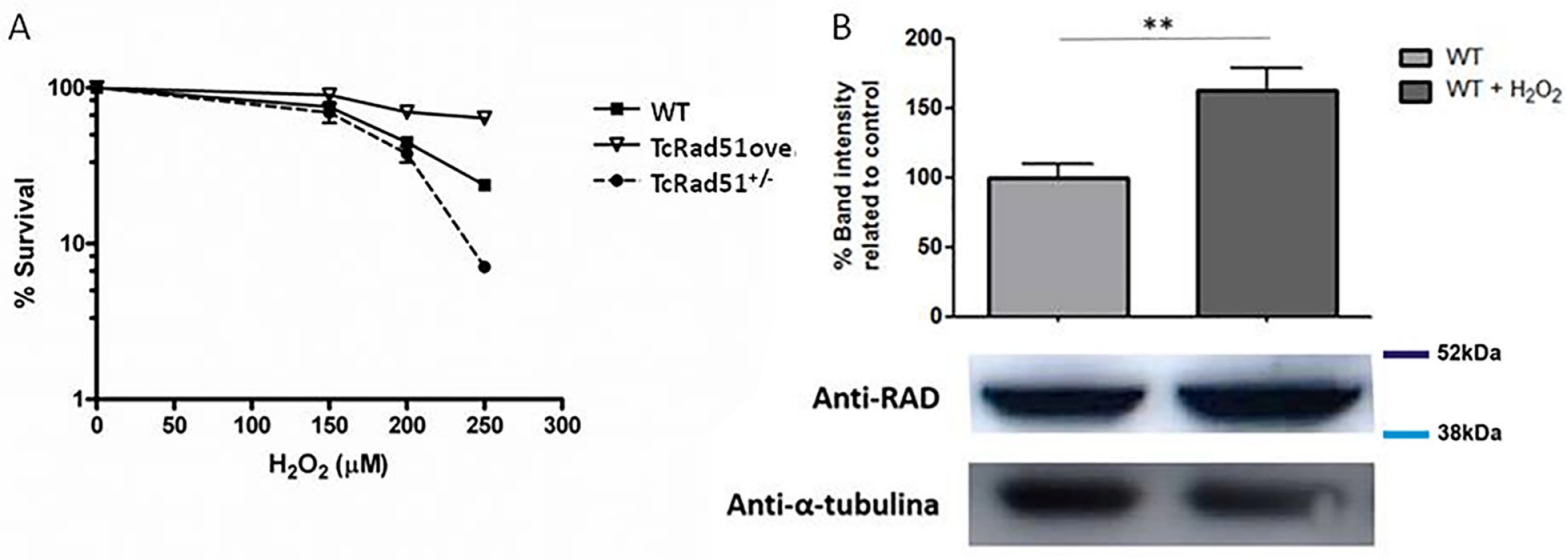
*T*. *cruzi* response to oxidative stress. (A) The sensitivity of WT, TcRAD51^ox,^ and TcRAD51^+/-^ cells against 150, 200 and 250 μM H_2_O_2_. Parasites were counted 48 h after treatment. Numbers are represented as a percentage of untreated cells. Values represent the mean of triplicates. Error bars indicate standard deviations. (B) Bar chart representing the detection of TcRad51 protein levels in epimastigotes extracts from WT cultures after treatment with 200 μM of H_2_O_2_ (performed in triplicate). Cell lysates were separated by SDS–PAGE and proteins were detected by Western blot with anti-TcRad51 (1:2,000) antiserum and peroxidase-conjugated anti-IgG secondary (1:10,000 or 1:12,000). A control showing tubulin levels was detected using mouse anti-Tubulin (1: 12,000) antiserum. Statistical analysis was performed using Student’s *t*-test. ** *p* < 0.01.

Different oxidative lesions can be promoted to DNA by H_2_O_2_, including DSBs [29]. DSBs can be formed by oxidation through aborted repair of primary DNA lesions, or through stalled replication forks. Since TcRad51 does not appear to be involved in the repair of crosslink DNA damage, we decided to evaluate the contribution of TcRad51 to the cellular response against oxidative stress. To address this possibility, cells were exposed to different doses of H_2_O_2_ and had their survival rates determined after 48 h following treatment measured. As shown in Fig 5A, TcRAD51^ox^ parasites had an increased resistance against H_2_O_2_ when compared to WT parasites, while TcRAD51^+/-^ cells were more sensitive to oxidation than WT parasites: in the presence of 250 μM H_2_O_2_, 24% of WT cells survived compared with 64% of TcRAD51^ox^ and 7% of TcRAD51^+/-^ cells. To test the involvement of TcRad51 in the response to H_2_O_2_ treatment, a Western blot was performed using anti-TcRad51 antibody. Fig 5B shows that the levels of TcRad51 increase after H_2_O_2_ treatment in WT cells. These results suggest that TcRad51 can mediate resistance against H_2_O_2,_ most likely by guiding HR in repairing the damage caused by this oxidant agent.

### TcRad51 levels influence the intracellular growth of amastigotes

**Fig 6:**
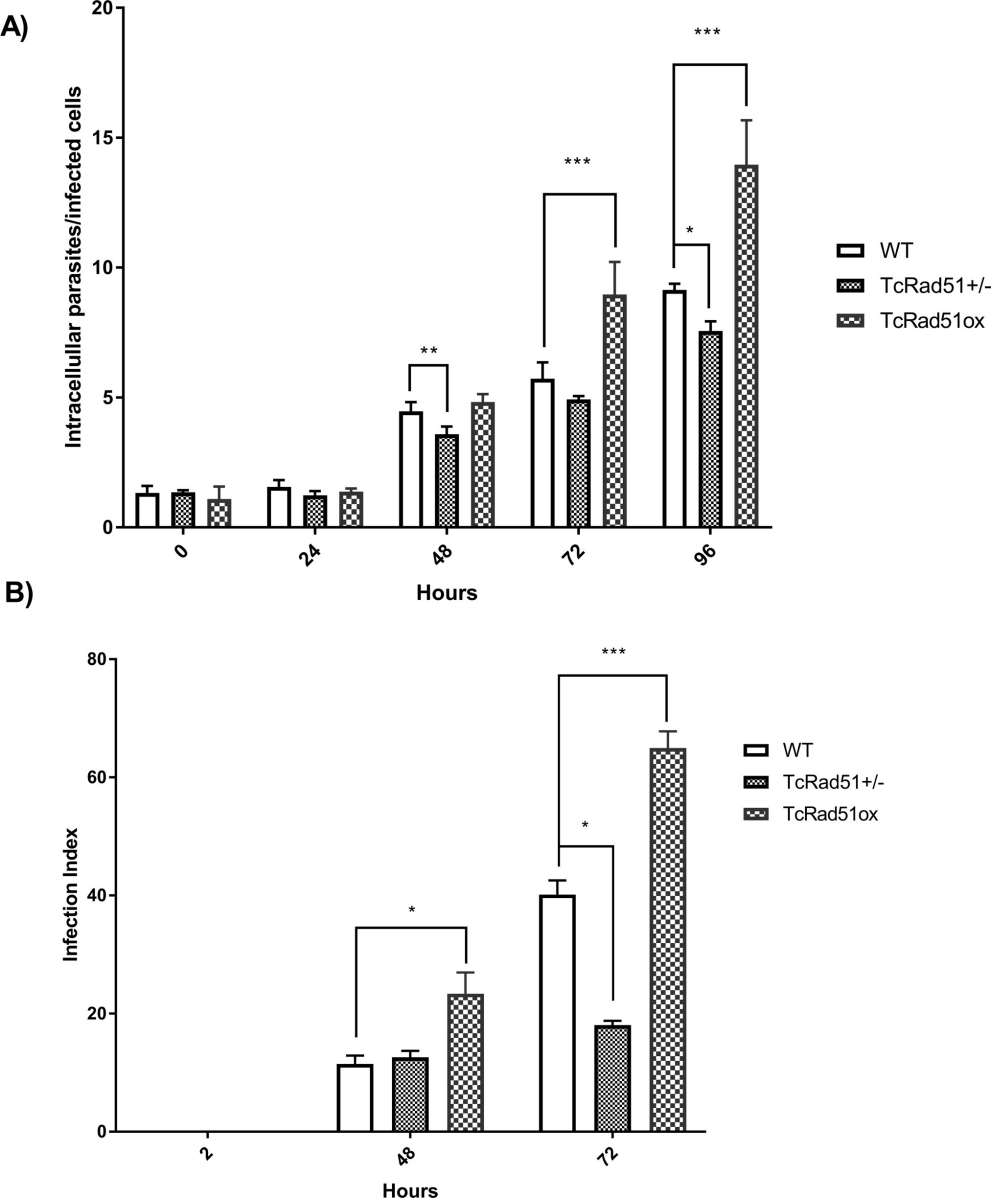
TcRAD51 expression levels are related to the intracellular growth of *T*. *cruzi* in fibroblasts and macrophage cells. Murine fibroblasts were exposed to trypomastigotes (MOI of 50) for 30 min. Monolayers were washed to remove extracellular parasites and either fixed (PFA 4%) or incubated with fresh medium without parasites for different times. Slides were stained by immunofluorescence and analyzed in a fluorescence microscope. A: Number of intracellular parasites per infected cell for WT, TcRAD51^+/-^ and TcRAD51^ox^ infected cultures ranging from 0 up to 96 h post-infection. Data were analyzed using two-way ANOVA test with Bonferroni post-test (***p < 0.001, **p < 0.01, *p < 0.05). At least 200 fibroblasts were analyzed. B: Macrophages obtained from the conversion of THP-1 monocytes into macrophages by the addition of PMA and incubated for 72 h were subjected to infection with WT, TcRAD51^+/-^ or TcRAD51^ox^ trypomastigotes (MOI of 15). Cells were washed to remove extracellular parasites, and either fixed or re-incubated with medium for 48 and 72 h. Slides were stained with Giemsa and analyzed to determine the infection index (percentage of infected macrophages multiplied by the average number of amastigotes per macrophage) for each parasite population. At least 300 macrophages were analyzed. Data were analyzed using two-way ANOVA test with Bonferroni post-test (***p < 0.001, *p < 0.05).

In order to investigate whether TcRad51 levels affect intracellular replication after the invasion of mammalian host cells, fibroblast cultures were infected with WT, TcRAD51^-/+^ or TcRAD51^ox^ cells. The number of intracellular parasites per infected cell was assessed after 24, 48, 72 and 96 h of infection. Fig 6A shows that, during the first 24 h of infection, WT, TcRAD51^+/-^ and TcRAD51^ox^ cells showed a similar pattern of infection, with an approximately equal number of intracellular parasites per infected cell. However, after 24 h of infection, when parasites differentiate to the amastigote form and replicate by binary fission, cells behaved differently. TcRAD51^+/-^ cells showed a significant decrease in growth when compared to WT or TcRAD51^ox^ cells 48 h postinfection (*p* < 0.01 and *p* < 0.001, respectively). Additionally, after 72 h of infection, an increased number of intracellular parasites could be observed in fibroblast cultures infected with TcRAD51^ox^ parasites (*p* < 0.001), while TcRad51^+/-^ cells maintained lower levels of growth; this differential growth pattern was also observed at 96 h of infection. This result demonstrates that TcRad51 levels and parasite intracellular development show a direct relationship as TcRAD51^+/-^ and TcRAD51^ox^ cells presented opposite behavior, reinforcing the importance of TcRad51 for parasite survival and growth in the intracellular environment.

The role of TcRad51 in the infection of macrophages was also studied (Fig 6B). Similarly to results observed in fibroblasts, overexpression of TcRad51 increased the rate of infection of *T*. *cruzi* in macrophages. After 48 and 72 h of infection, the number of cells infected with TcRAD51^ox^ cells was higher compared to WT and TcRAD51^+/-^. Also, the infection rate of macrophages with TcRAD51^+/-^ parasites remained low even after 72 h of incubation. These observations confirm results obtained for fibroblast showing that TcRad51 exhibit important roles during *T*. *cruzi* infection.

### TcRad51 levels impact DNA repair and intracellular replication after gamma irradiation in *T*.*cruzi*

**Fig 7:**
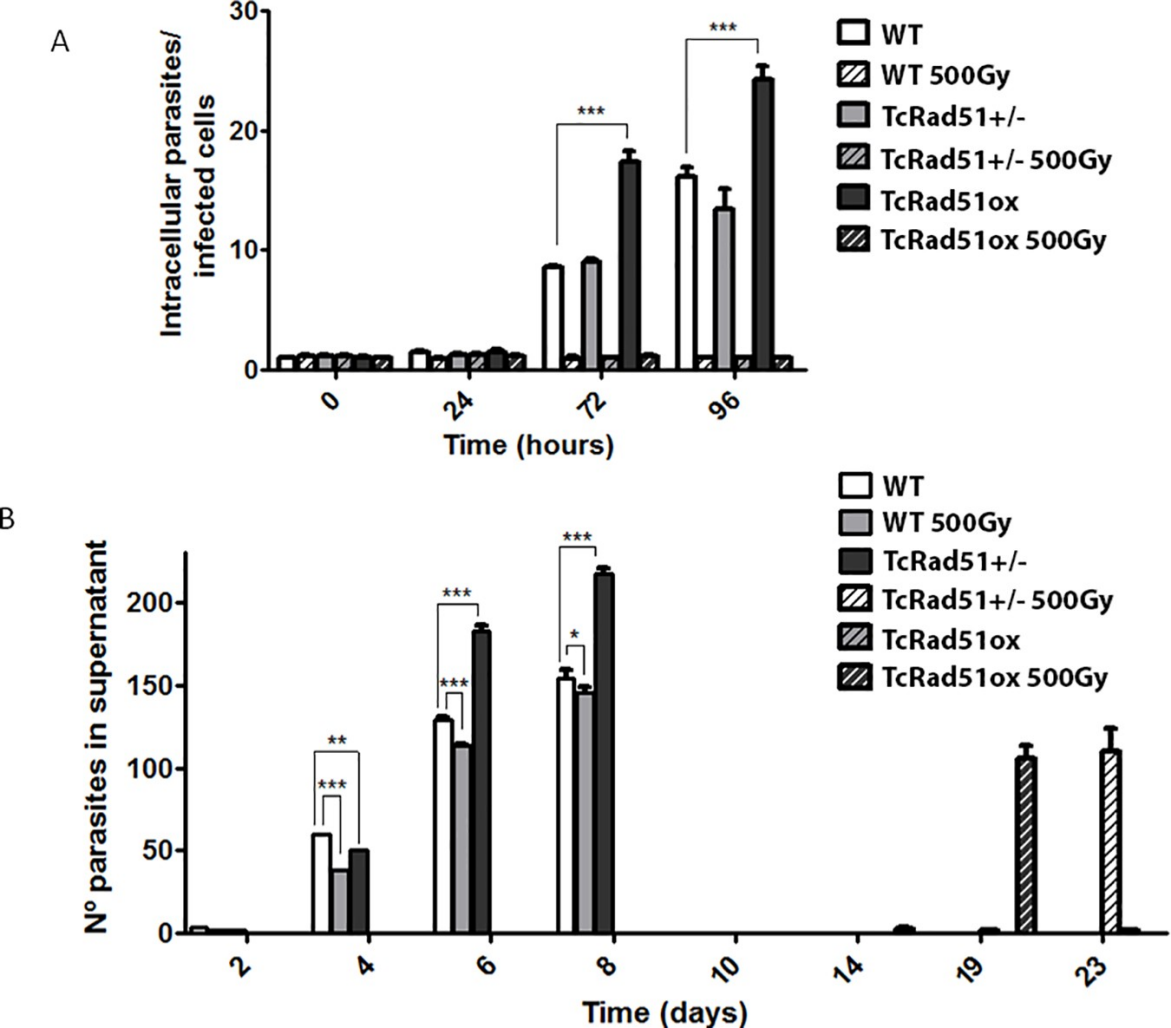
The Intracellular growth of *T*. *cruzi* after gamma irradiation are related to TcRAD51 expression levels. (A) The number of intracellular parasites in cultures infected with WT, TcRAD51^+/-^ and TcRAD51^ox^ parasites treated or not with 500 Gy of gamma radiation over time. (B) Kinetics of trypomastigote release in cultures infected with WT, TcRAD51^+/-^ and TcRAD51^ox^ parasites treated or not with 500 Gy of gamma irradiation. The number of WT, TcRAD51^+/-^ and TcRAD51^ox^ trypomastigotes in the cellular supernatant after fibroblast exposure to gamma radiation was monitored for 23 d. Data were analyzed using two-way ANOVA test with Bonferroni post-test (***p < 0.001,**p < 0.01, *p < 0.05).

In order to evaluate the expression levels of TcRAD51 in genetically-modified amastigotes, WT, TcRAD51^+/-^ and TcRAD51^ox^ parasites were exposed to 500 Gy of gamma irradiation prior to fibroblast infection. Gamma radiation treatment precluded parasite replication inside fibroblasts for the next 96 h after treatment (Fig 7A). We also analyzed the number of trypomastigotes released from fibroblasts over time. Our results show that TcRAD51^ox^ trypomastigotes release is higher than that verified for WT trypomastigotes. On the other hand, no trypomastigote release was observed for gamma irradiated TcRAD51^+/-^ infected cultures during the period of 23 d of infection, after which the cells became unviable (Fig 7B). These results suggest that our genetically-modified cells do not cease to synthesize different quantities of TcRad51 in amastigote form and that the phenotype observed in amastigotes is similar to the epimastigote phenotype after gamma irradiation.

### Levels of TcRad51 affect *T*. *cruzi* infection in mice

**Fig 8:**
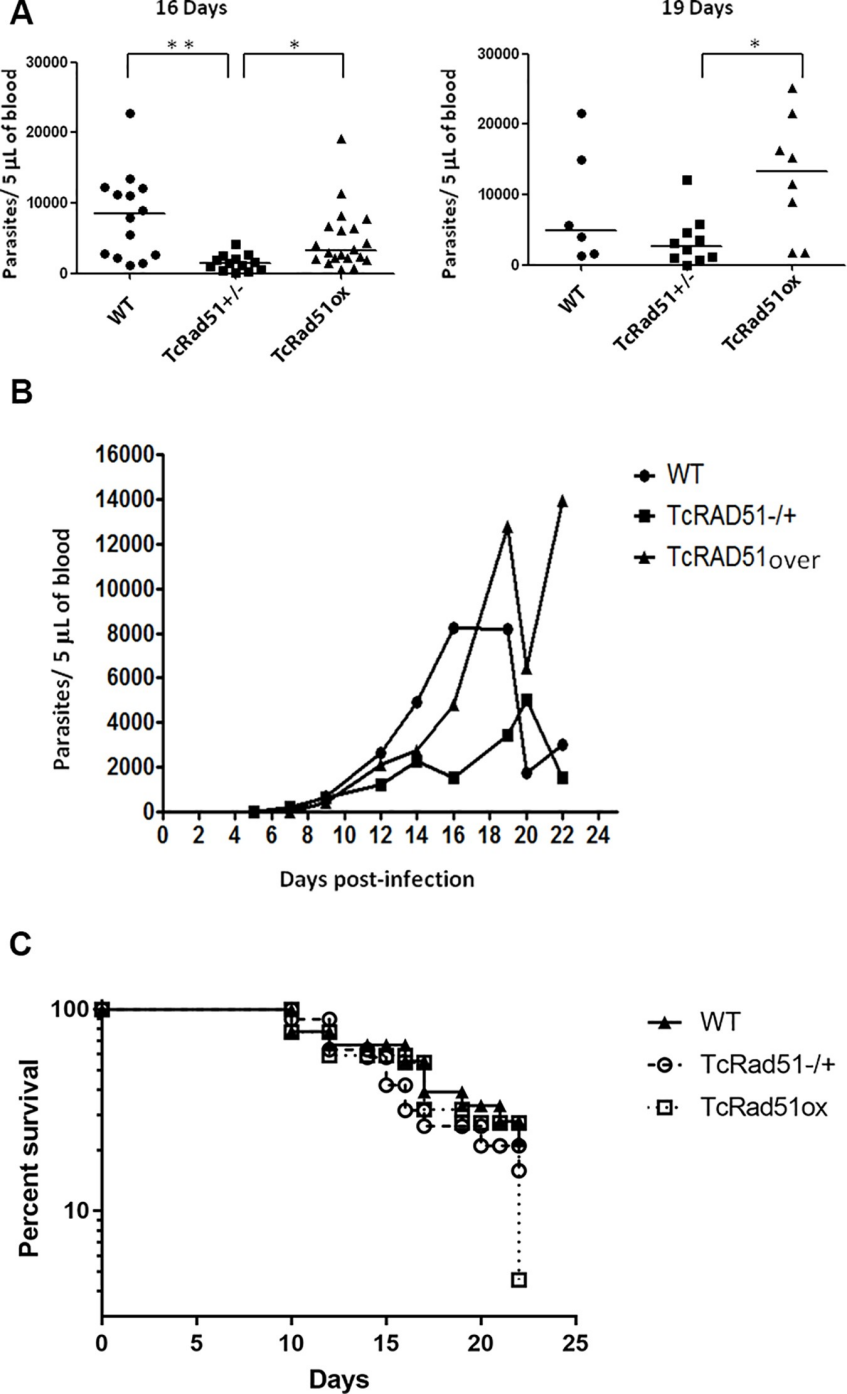
Parasitemia from animals infected with WT, TcRAD51^+/-^ or TcRAD51^ox^ parasites. Male Swiss mice were intraperitoneally infected with 5,000 bloodstream forms. Parasitemia was evaluated from the 5^th^ to the 22^nd^ day post-infection by counting blood trypomastigotes collected from mouse’s tail. (A) Parasite numbers of each mouse at days 16 and 19 after infection are shown. Horizontal lines indicate the mean of parasite numbers from each *T*. *cruzi* sample infecting mice. Differences between groups were determined using Kruskal–Wallis test followed by Dunn post hoc test (16th day), or ANOVA test followed by Tukey post hoc test (19th day). Statistically different values are indicated as follows: *p < 0.05, **p < 0.001.(B) Parasitemia curves from animals infected with CL Brener WT, TcRAD51^-/+^ or TcRad51^ox^ parasites. Each curve represents the mean of 2 distinct experiments (*n* = 19 to 22 mice per *T*. *cruzi* group). The mice were infected with 5,000 blood trypomastigotes intraperitoneally. Parasitemia was evaluated from the 5^th^ to the 22^nd^ day after infection. (C) Survival of infected animals exposed with three different strains of *T*. *cruzi*: WT, TcRad51^ox,^ and TcRad51+/-. Male 3-week old Swiss mice were infected intraperitoneally with 5,000 TCT’s. The mortality is presented as a percentage when compared to the control group (mice were injected with saline solution). No statistical difference were observed at the times analyzed, although the overexpressor strain was able to achieve a high mortality at the end time of the experiment.

In order to evaluate the role of TcRad51 during animal infection, mice were intraperitoneally inoculated with 5,000 *T*. *cruzi* bloodstream forms of CL Brener WT, TcRAD51^+/-^ or TcRAD51^ox^ cells. As shown in Fig 8A and 8B, the peak of parasitemia in mice infected with WT parasites was verified from the 16^th^ to the 19^th^ day after the infection, as described earlier [30]. Infection of TcRAD51^ox^ cells presented a significant delay in its peak, which happened on the 19^th^ day after infection; however, infection with TcRAD51^ox^ cells produced a higher number of parasites compared with mice infected with TcRAD51^+/-^ (*p* < 0.05). Mice infected with TcRAD51^+/-^ showed a decreased peak of parasitemia at 20 d post-infection (Fig 8B). Together, these results show the importance of TcRad51 for the success of *T*. *cruzi* in infecting the mammalian host.

In addition, mortality rates of mice infected with different *T*. *cruzi* samples (WT, TcRAD51^+/-^ or TcRAD51^ox^) were evaluated from the 10^th^ to the 22^nd^ day after infection. Data were analyzed by Fisher’s exact test, and no statistically significant difference was observed among the samples analyzed (Fig 8C).

### TcRad51 levels affect *T*. *cruzi in vivo* resistance against benznidazole

We previously showed that epimastigote cells overexpressing TcRad51 display increased *in vitro* resistance against benznidazole when compared to WT [31]. To verify whether TcRad51 levels affects *T*. *cruzi in vivo* resistance against benznidazole, parasite’s susceptibility to this drug was determined in groups of mice intraperitoneally infected with CL Brener WT, TcRAD51^ox^ or TcRAD51^+/-^ parasites. After 5 d of infection, animals became subject of a 20-day treatment with benznidazole and had blood samples collected for hemoculture. Percentage of cure was analyzed through the presence of parasites. Table 1 shows the percentage of cure induced by benznidazole after long-term treatment of different groups of infected mice. The percentage of cure in mice group infected with WT *T*. *cruzi* was 92.6%. In contrast, mice infected with TcRAD51^ox^ parasites presented a 50% cure. Then, TcRAD51 overexpression conferred an increase of over 40% in resistance against benznidazole (*p* < 0.05). On the other hand, no statistically significant difference between the percentage of cure between WT and TcRAD51^+/-^ parasites was observed. According to the literature, hemoculture is the best indicator of cure for mice after chemotherapy, and it has been used for analyzing susceptibility *in vivo* of *T*. *cruzi* strains to benznidazole [32].

**Table 1:** Percentage cure induced by benznidazole after long-term treatment of mice infected with Cl Brener wild-type, TcRAD51^-/-^ or TcRAD51^ox^ cells.

*T*.*cruzi samples infecting mice*	Number of mice	Number of positive mice	Percentage cure
**WT**	14	1	92.6
**TcRAD51+/-**	14	2	85.7
**TcRAD51over**	12	6	50

*p < 0.05 *vs*. mice infected with wild-type parasites. Data were analyzed by Fisher’s exact test.

Male Swiss mice at 3 weeks of age were intraperitoneally infected with 5,000 *T*. *cruzi* bloodstream forms. Five days after infection, blood samples of these animals were analyzed, and positive mice were subjected to treatment with benznidazole for 20 consecutive days. Thirty days after treatment, blood cultures were performed. Samples were incubated in BOD greenhouse and analyzed for positivity after 30 and 60 d.

## Discussion

Rad51, a strand-exchange protein, is a central player in HR [1]. In this work, we first knocked out one allele of TcRAD51 and examined the phenotypic effects of this mutation. Levels of TcRad51 have important consequences for growth and infection of *T*. *cruzi*. We also showed that TcRAD51^+/-^ parasites presented a delayed growth recovery after gamma radiation exposure, in oppose to TcRAD51^ox^ parasites, which recover faster than the WT ones. Second, the same behavior is observed for damage induced by H_2_O_2_. These two effects are relevant as levels of TcRad51 correlate with growing capacity and infectivity of parasites in mammalian cells, as well as in the animal model, suggesting that HR is required for parasite infectivity.

We also verified that the deletion of one allele of TcRAD51 is sufficient to reduce the rates of TcRad51 foci formation after irradiation, as determined by immunolocalization through fluorescence quantification (Fig 4). Indeed, TcRad51 accumulates in the nucleus after irradiation, initially as foci. The inability to distinguish discreet foci at later time points after irradiation may be due to their increased number and intensity beyond the limits of microscopic detection as protein levels increase, which is consistent with the fact that TcRAD51^+/-^ cells present lower levels of TcRAD51 expression and also showed a delay in foci formation in the nucleus after radiation treatment. Most likely, these data explain the behavior of *T*. *cruzi* after gamma irradiation, with lowered levels of TcRad51 in the TcRAD51^+/-^ cells causing a delay in DNA repair activity, which will interfere in the time parasites take to overcome growth arrest caused by exposure to ionizing radiation. On the other hand, the higher levels of TcRAD51 expression observed in TcRAD51^ox^ cells, before and after gamma irradiation, can be correlated with a faster both DNA repair and growth recovery after radiation exposure. The presence of TcRad51 even after the repair of chromosomes verified through PFGE (Fig 2) suggests that not all lesions are repaired within 24 h, leading to the observation of populational cellular arrest for longer times. These results confirm that TcRad51 is directly involved in HR for DNA repair. Since *T*. *cruzi* appears to lack key proteins involved in NHEJ [8], a reaction that has never been described *in vitro* [9] or *in vivo* for other trypanosomes [10,11], HR involving TcRad51 appears to be the pathway that confers resistance to high levels of gamma radiation in *T*. *cruzi*, as proposed around 30 years ago [7,33]. Nonetheless, the exact recombination strategy *T*. *cruzi* uses to reassemble its chromosomes, and the reason why this parasite needs such a pathway, require further examination.

We also evaluated the contribution of TcRad51 to the repair of lesions caused by cross-linking agents such as UV light and cisplatin. HR involvement in repairing damage promoted by these agents–which are capable of blocking the replication machinery in other organisms–has been documented [34–37], including a critical role in the regeneration of active replication forks at blocking lesions [38,39]. Interestingly, sensitivity to either UV light or cisplatin were not altered by changing TcRAD51 expression, indicating that TcRad51, and therefore HR, does not play a major role in the repair of lesions caused by these genotoxic agents. In fact, cisplatin forms intra- and inter-strand cross-links, while UVC light mostly causes intra-strand cross-links–primarily cyclobutane pyrimidine dimers and pyrimidine 6–4 pyrimidone photoproducts [36]. These lesions in DNA can block essential cellular process other than replication, such as transcription [40,41]. In this scenario, the stalling of RNA polymerase triggers the activation of transcription-coupled repair (TCR), which removes lesions located on the transcribed strand of active genes [42]. The genome of *T*. *cruzi* is highly unusual among eukaryotes, being transcribed into long multigene transcripts with relatively few non-transcribed regions [8]. It seems likely, therefore, that the majority of these lesions will cause a severe block to transcription, and they must be recognized and repaired by TCR. Indeed, recently Machado and colleagues demonstrated, through RNA interference of TCR components in *T*. *brucei* play a major role in the repair/tolerance of UV lesions and repair of cisplatin lesions [43].

Although UV and cisplatin may generate replicative stress, the importance of TcRad51 in resolving this cellular state is still unknown. Two hypotheses may explain this observation: the first one relies on the possibility of replication stress generated by these treatments being resolved by other repair pathways; the second one is that the remaining TcRad51 synthesized by TcRAD51^+/-^ cells suffice to effectively deal with this insult. In fact, TcRad51 is not involved in all types of replicative stress response since we did not observe any phenotypic difference after HU treatment (Fig 2C); however, TcRAD51^-/+^ parasites are also more sensitive to MMS (Fig 2D). This difference could be due to the lack of DNA damage in HU-treated cells, whereas MMS generates DNA lesions that could not be passed by replicative DNA polymerases. In fact, using an antibody against gamma histone 2A, a marker for DNA double-strand breaks [21], we were able to observe an increased number of DNA lesions in TcRAD51^-/+^ cells after with MMS, but not after HU treatment when compared to WT cells (Fig 2E and 2F). Therefore, TcRAD51 may be involved only in some types of replicative stress [44].

Thus, cross-link lesions may be repaired or tolerated before DNA replication, meaning that TcRad51 would be minimally involved in the repair. In this regard, studies in *T*. *brucei* have shown that homozygous mutants of RAD51 are similarly unaffected in sensitivity to UV light lesions [6] or cisplatin (S4 Fig), suggesting that the strategy for tackling cross-link damage may be kinetoplastid-wide.

Although TcRad51 does not appear to contribute to cross-link repair, we showed that this protein is important to deal with the oxidative damage. Overexpression of TcRAD51 increases resistance against H_2_O_2_, while the deletion of one allele of TcRAD51 increases sensitivity to this oxidant compound (Fig 6). H_2_O_2_ is an oxidative agent that primarily causes DNA base damage, including single-strand breaks, base loss and the generation of 7,8-dihydro-8-oxoguanine (8oxoG) [45,46]. Treatment with H_2_O_2_ increases TcRad51 protein level in WT cells, which reinforces the importance of the HR repair pathway in response to oxidative stress. Many studies, as reviewed in [47], have reported that one of the main lesions produced by H_2_O_2_, 8-oxoguanine, blocks the transcription machinery only in a few specific DNA sequence contexts that cause helix distortion. Thus, TCR is unlikely to be activated by oxidative damage and not repaired until S phase, when it will block the replication fork. This stalled replication fork can be a substrate for TcRad51, and this protein will allow HR to repair DNA damage so that DNA replication can be resumed. This repairing reaction is necessary to eliminate lesions that impede fork progression, in order to avoid fork collapse and allow forks to resume and complete chromosome replication [48,49]. Therefore, it seems likely that TcRad51 mediates the resistance against oxidative damage to DNA through the repair of DNA damage during replication. The data we provide here on TcRad51 add to a growing body of evidence that trypanosomatids use many pathways are needed to cope with oxidative damage, including some other repair pathways, most notably mismatch repair [50,51].

Since TcRad51 is important for *T*. *cruzi*‘s response to oxidative stress to which this parasite is exposed during its life cycle, the relevance of TcRad51 and the HR pathway for *T*. *cruzi* both intracellular replication and survival inside the cell host was also evaluated in this work. Aiming to confirm that genetic modifications of TcRAD51 (i.e, single allele knock out and overexpression) would be stable in the amastigote form, we irradiated trypomastigote forms before the infection, and then we followed the growth of the amastigotes in fibroblast cells. Irradiated parasites showed a similar phenotype to that one observed for epimastigote forms, i.e, a delay in the cellular growth. Additionally, TcRAD51^ox^ parasites recovered growth faster than WT cells. Non-irradiated TcRAD51^ox^ parasites also presented enhanced multiplication inside fibroblasts, and TcRAD51^+/-^ parasites showed decreased growth when compared to WT.

After confirming the stability of the genetic modifications of TcRAD51, we analyzed the infectivity of the modified cells in fibroblast and macrophage cells. In these both types of cells, we verified that TcRAD51^ox^ parasites exhibited an increased growth and that TcRAD51^-/+^ showed a decreased growth when compared to WT cells; however, these differences are more accentuated in macrophage cells. The role of TcRad51 in intracellular multiplication could be attributed to DNA repair in response against oxidative lesions. Gupta and colleagues [52] demonstrated that, during *T*. *cruzi* invasion and intracellular growth in cardiomyocytes, an exacerbated reactive oxygen species production occurs in the host cell cytoplasm, being detected 2 h post-infection, and showing an exponentially increase until 48 h post-infection, fact that correlates with our findings that growth differences between the *T*. *cruzi* strains were observed after 48 h of infection. Further evidence is provided by examination of a *T*. *cruzi* that overexpress the TcMutT gene, which results in an increase in the number of amastigotes, which occurs with the same kinetics seen for TcRAD51^ox^ parasites [30]. MutT is responsible for removing 8-oxo-dGTP from the nucleotide pool; this lesion is considered particularly deleterious because it could generate DNA double-strand breaks if it is not properly repaired [53]. The increased TcRAD51^+/-^ sensitivity in macrophages could be related to the level of oxidative stress produced by this kind of cell when compared to fibroblasts. The curve in the presence of H_2_O_2_ (Fig 5) showed that TcRAD51^+/-^ parasites are more sensitive than WT ones to this drug only in high doses, which suggests that the level of TcRad51 in TcRAD51^-/+^ is sufficient to deal with the damage caused by low doses of oxidative stress.

The importance of TcRad51 to infection was not limited to *in vitro* models. Although TcRAD51^ox^ parasites show a delay in the peak of parasitemia in mice compared to WT cells, all the mice infected the TcRAD51^ox^ *T*. *cruzi* presented an increased number of parasites in the bloodstream, most likely in consequence of increased intracellular replication. Moreover, TcRAD51^+/-^ parasites showed lower parasitemia throughout, and no parasitemic peak, indicating that loss of only a single allele is detrimental to *in vivo* survival. Interestingly, although infection with TcRAD51^+/-^, TcRAD51^ox^, and WT *T*. *cruzi* led to different levels of parasites in the bloodstream of mice (Fig 8A and 8B), infected animals did not exhibit a parasitemia-dependent rate of mortality (Fig 8C), showing that parasitemia itself does not determine host mortality. Finally, recent work from our group that showed that TcRad51 could be involved with the resistance to benznidazole *in vitro* [31]. In fact, we demonstrated here that TcRad51 is also involved in resistance to this drug *in vivo*, since overexpression of TcRAD51 caused an increased resistance to benznidazole, decreasing the rate of cure in mice infected with TcRAD51^ox^ parasites. It is possible that benznidazole could generate 8-oxo-dGTP and, as described above, an inappropriate repair of this lesion could generate a DSB that will be substrate to the HR pathway. Therefore, TcRad51 could be an important protein for *T*. *cruzi*’s metabolism, since it is well established that HR repair becomes very efficient if a template is present when compared to MMEJ [54–56].

In summary, our results suggest that TcRad51, and thereby HR, has a pivotal role in *T*. *cruzi* genome maintenance in a number of stages of the parasite life cycle. Moreover, this key protein for HR is involved in the repair of DSBs and oxidative lesions in *T*. *cruzi*, being crucial for parasite’s survival and persistence during mammalian infection.

## Supporting information

S1 Fig DNA sequencing containing ~700pb, which includes the beginning of the hygromycin gene, the 500pb region upstream of the Rad51 gene and the region beyond the cloned cassette. The comparison is made between the remaining wild-type allele, the annotated sequence in the TrytripDB database and the Hygromycin gene used in the construction. In blue: the upstream region to the top of the vector used in cloning. In red: the beginning of the coding sequence of Rad51. In green: the start of the hygromycin phosphotransferase sequence.
(TIF)

S2 Fig A) *T. cruzi* growth curve after treatment with 30 mM HU. B) *T. cruzi* growth curve after treatment with 10 mM HU. For both panels, the arrow indicates the point when the drug was removed.
(TIF)

S3 Fig Kinetics of nuclei-localized TcRad51 in WT, TcRAD51^-/+^, and TcRAD51^ox^ cells are shown. TcRad51 was detected using anti-TcRad51 antibody raised in mouse (diluted 1:2,000) and visualized with Alexa 555 conjugated goat-derived anti-mouse IgG secondary (diluted 1:5,000). DNA is shown stained with DAPI (blue).
(TIF)

S4 Fig Sensitivity of WT and TbRAD51^-/-^ cells to 1.7, 3.3 and 5 μM of cisplatin. Parasites were counted 48 h after treatment. Numbers are represented as a percentage of untreated cells. Values represent the mean of triplicates. Error bars indicate standard deviations.
(TIF)
