# Supplementary figures and images for: The in vivo and in vitro roles of Trypanosoma cruzi Rad51 in the repair of DNA double strand breaks and oxidative lesions
Source: PLoS Negl Trop Dis. 2018 Nov 13;12(11):e0006875. doi: 10.1371/journal.pntd.0006875 (PMC6258567; doi:10.1371/journal.pntd.0006875)

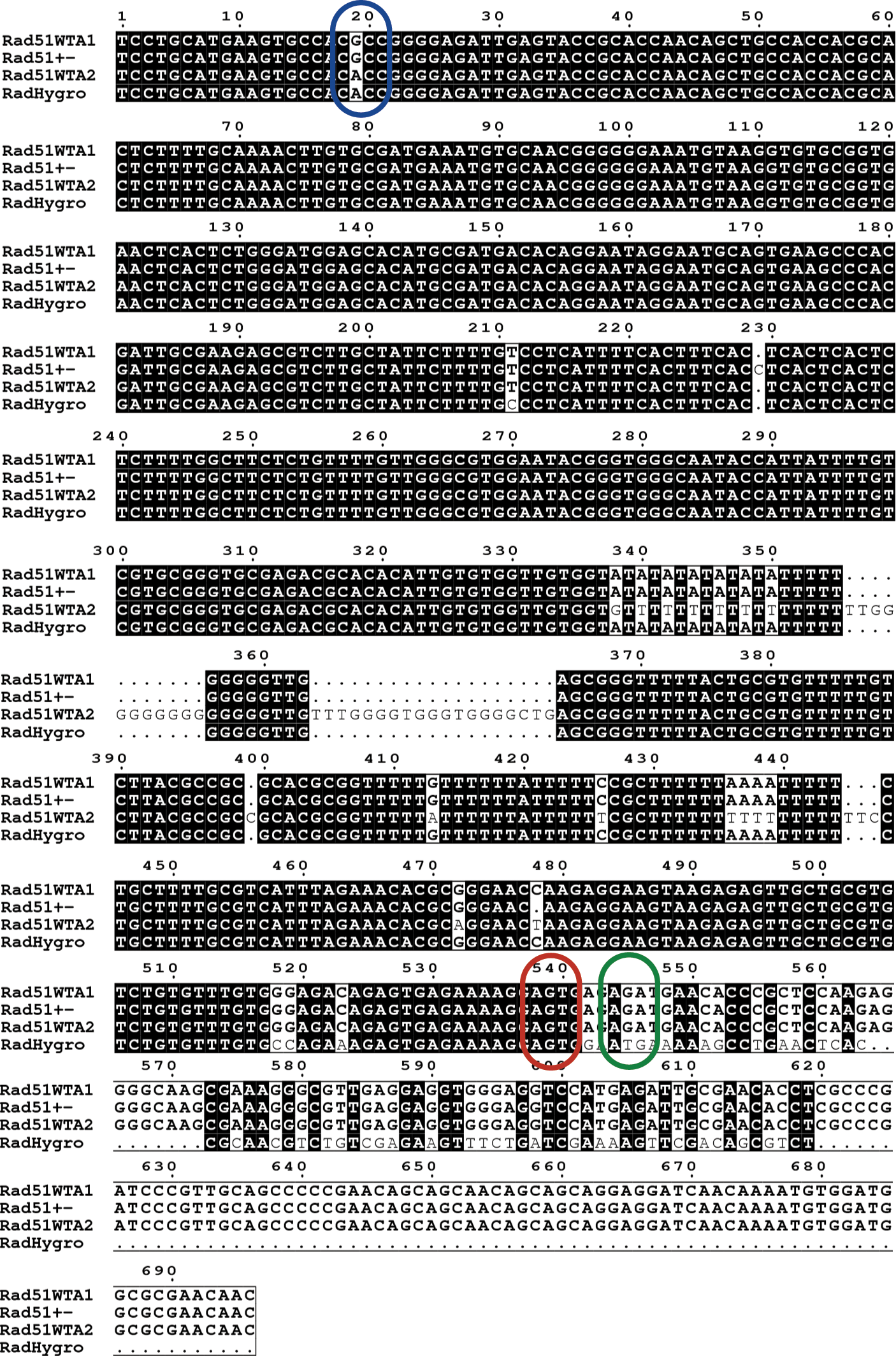

Supplement: S1 Fig — The comparison is made between the remaining wild-type allele, the annotated sequence in the TrytripDB database and the Hygromycin gene used in the construction. In blue: the upstream region to the top of the vector used in cloning. In red: the beginning of the coding sequence of Rad51. In green: the start of the hygromycin phosphotransferase sequence. (TIF) [file pntd.0006875.s001.tif]

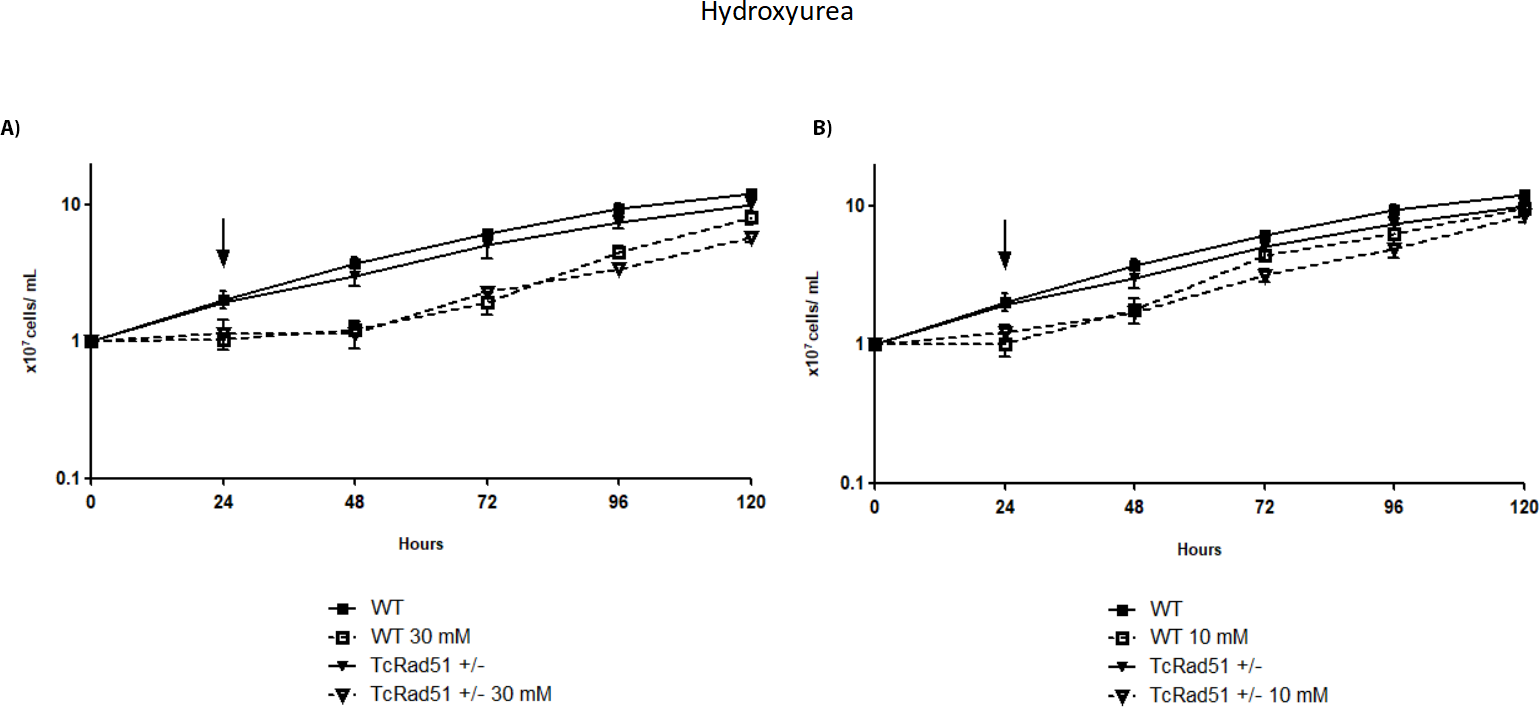

Supplement: S2 Fig — A) T. cruzi growth curve after treatment with 30 mM HU. B) T. cruzi growth curve after treatment with 10 mM HU. For both panels, the arrow indicates the point when the drug was removed. (TIF) [file pntd.0006875.s002.tif]

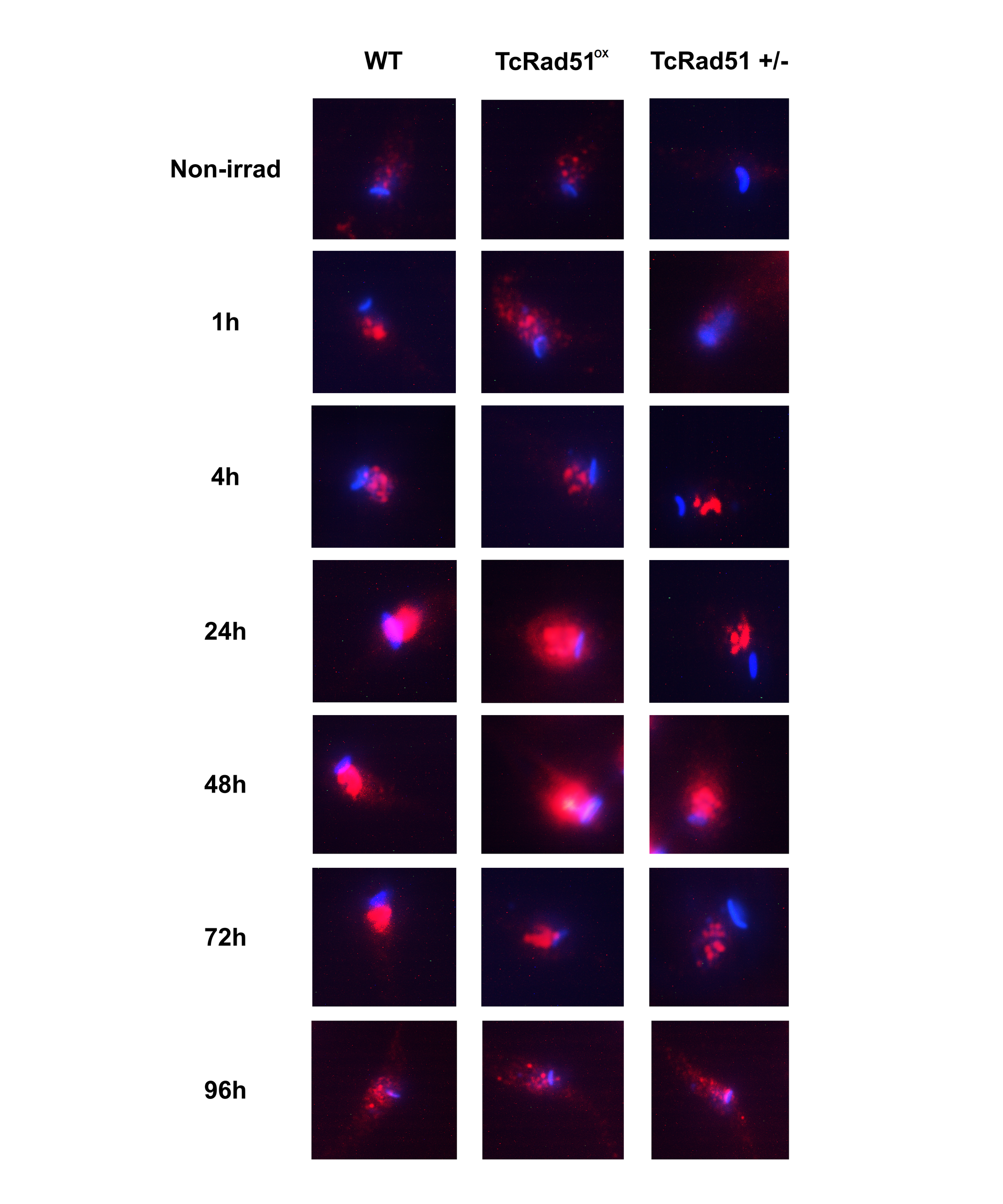

Supplement: S3 Fig — TcRad51 was detected using anti-TcRad51 antibody raised in mouse (diluted 1:2,000) and visualized with Alexa 555 conjugated goat-derived anti-mouse IgG secondary (diluted 1:5,000). DNA is shown stained with DAPI (blue). (TIF) [file pntd.0006875.s003.tif]

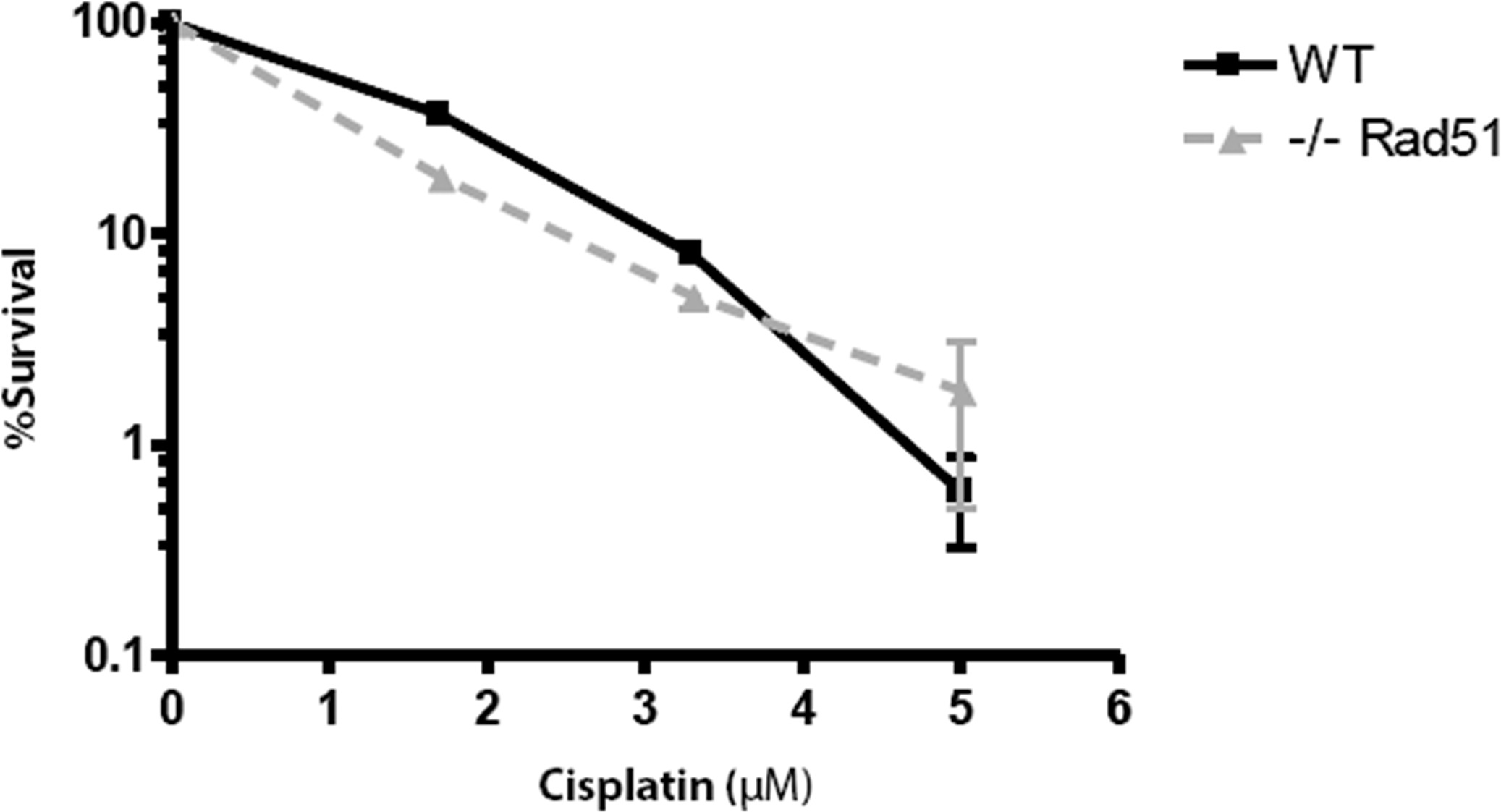

Supplement: S4 Fig — Parasites were counted 48 h after treatment. Numbers are represented as a percentage of untreated cells. Values represent the mean of triplicates. Error bars indicate standard deviations. (TIF) [file pntd.0006875.s004.tif]
